# Stress, Professional Burnout, and Employee Efficiency in the Greek National Organization for the Provision of Health Services

**DOI:** 10.3390/clinpract13060135

**Published:** 2023-11-25

**Authors:** Alexandra Vlassi, Evangelos Vitkos, Despoina Michailidou, Panagis M. Lykoudis, Lambrini Kioroglou, Athanassios Kyrgidis, Ioannis Tilaveridis, Theodoros Dardavesis

**Affiliations:** 1 Laboratory of Hygiene, Social & Preventive Medicine and Medical Statistics, Department of Medicine, School of Health Sciences, Aristotle University of Thessaloniki, 541 24 Thessaloniki, Greece; alessandra.vlassi@gmail.com (A.V.);; 2 Department of Oral and Maxillofacial Surgery, University Hospital of Crete, 715 00 Heraklion, Greece; 3 Department of Oral and Maxillofacial Surgery, George Papanikolaou General Hospital, 570 10 Thessaloniki, Greece; 4 Consultant Hepato-Pancreato-Biliary Surgeon Honorary Lecturer, Division of Surgery & Interventional Science, University College London, London NW3 2PS, UK; 5 School of Medicine and School of Law, Aristotle University of Thessaloniki, 541 24 Thessaloniki, Greece; 6 Department of Oral and Maxillofacial Surgery, Aristotle University of Thessaloniki, 541 24 Thessaloniki, Greece

**Keywords:** anxiety, burnout, wellness, workplace errors

## Abstract

Background: Workplace stress and burnout in the Greek healthcare system had been considered severe even before the high pressure of the COVID-19 pandemic. We aimed to investigate occupational quality of life and burnout effects on workplace errors among the administrative staff in the Greek healthcare system. Methods: We enrolled 120 administrative healthcare employee participants between April and May 2019. Occupational burnout was assessed using the Maslach Burnout Inventory—Human Services Survey and the Hospital Anxiety and Depression Scale. Findings: Inadequate staffing, a low sense of well-being, exhaustion, and low family income were associated with workplace errors. Increased workload and staff shortages were associated with occupation related quality of life. Conclusions: Targeted interventions supporting healthcare staff mental health are warranted. Application to Practice: Wellness and professional burnout can affect professional efficiency and are associated with workplace errors in the healthcare sector. Targeted interventions are warranted to support the mental health of healthcare staff during work and to prevent incidents of post-traumatic stress. Shortages of staffing may lead to an increase in the cost of the provided services.

## 1. Background

Healthcare worker burnout can have significant repercussions on the healthcare system and patients’ welfare [1]. Medical professionals play a crucial role in diagnosing, treating, and supporting patients; however, the demanding nature of their responsibilities, coupled with long hours and emotional strain, can lead to burnout. Stress and professional burnout in health professionals are essential factors that affect their everyday satisfaction that derives from their job and their efficiency [1,2]. In Greece, during the last decade, the financial crisis has imposed an additional burden on employees, at both the personal and professional levels. Regarding public healthcare, a substantial staff shortage is recorded, further burdening individuals and complicating the work of related professionals [3].

Previous studies showed that healthcare workers experience amplified workplace burnout during health emergencies, such as the recent pandemic [4]. The pandemic is also responsible for increased global healthcare demand. Relevant research showed that the demand for specialized healthcare would exceed capacity, burdening staff even more [5,6]. Moreover, workplace burnout is related to job stress, time pressure, limited organizational support, and high workload [7]. Other recent studies report that health workers in Greece, due to the organization of the healthcare system, are experiencing high levels of overall burnout and emotional exhaustion [8,9]. These characteristics are representative of healthcare demand in Greece. The demand for healthcare was reported to be rising, even during the two lockdowns that were imposed [2].

Wellness, quality of life, and well-being: all terms refer to the positive, perceived state of a person. Wellness is not just the absence of disease; rather, it is a separate positive state. Wellness is defined as a spectrum; on one end—high wellness—the individual is extremely happy; on the other end—low wellness—the individual is under intense stress and experiences depression [10]. Wellness can be measured with several tools, such as the Hospital Anxiety and Depression Scale [11] and the General Health Questionnaire [12]. The concept of burnout was originally conceived by health professionals; it is a condition of critical exhaustion—in contrast to chronic organizational stress—that leads to feelings of both workplace and emotional exhaustion [13], depersonalization, and a reduced degree of personal achievement [14].

Similar research has been conducted regarding the correlation between professional burnout and the wellness of health professionals (nursing staff and physicians) and their patients’ safety [15,16,17,18,19]. Most studies have concluded that both professional burnout and stress are related to an increased number of errors. Previous studies showing a significant correlation [20] used objective criteria to assess errors, thus suggesting a potential lack of sensitivity [15,17,18]. The responsibilities of the medical, nursing, and administrative staff of the Greek National Organization for the Provision of Health Services include the coordination of healthcare funds, the assignment of healthcare need contracts, and limiting expenses and allocating limited resources to a greater pool of unmet needs, thereby introducing the requirement for the staff to decide which needs will not be met.

This study aimed to investigate the way stress and professional burnout affects medical, nursing, and administrative staff of the Greek National Organization for the Provision of Health Services. Furthermore, we examined the impact of wellness and professional burnout on professional efficiency and their relation to workplace errors. Moreover, we investigated the participants’ departments’ staffing sufficiency (a shortage of staff is defined as either a vacancy of at least 30% of the department’s positions or that an excessive workload could not be dealt with by the existing staff without serious delays) and its relation to workplace errors.

## 2. Methods

### 2.1. Planning

Following Aristotle University of Thessaloniki institutional review board approval 4.95 17/07/2019, we conducted a cross-sectional survey between April and May 2019; data were recorded for 120 employees of the Auditing Agency for Social Security Funds, the Departmental Boards of the Greek National Organization for the Provision of Health Services, and the Healthcare Districts employees. The questionnaires were handed out to the employees outside working hours and were completed voluntarily, while no financial motive or compensation was given. Participants were given the opportunity to withdraw/revoke their consent at any time with no further explanation. Participant anonymity and confidentiality were ensured. Written informed consent was provided by all participants. The aim of the study [21] was made clear and participants were informed that it would take no more than 15 min to fill in the questionnaires. All procedures performed in studies involving human participants were in accordance with the ethical standards of the university’s Ethics Committee and with the 1964 Helsinki Declaration and its later amendments or comparable ethical standards.

### 2.2. Survey Sample

The target population of the survey was the medical, nursing, and administrative staff of the Greek National Organization for the Provision of Health Services. Our study sample constituted 50% of the employees of the selected departments. The sample of the survey included 120 participants. 

### 2.3. Data Collection

We used the Maslach Burnout Inventory MBI-HSS scale [14], following relevant approval, to record the employees’ degree of professional burnout and wellness. It includes 22 questions measuring emotional burnout, depersonalization, and personal fulfillment. It is a valid, and easy-to-use tool, and it has been officially translated into Greek [22] and presents Cronbach’s alpha reliability ranging from 0.7 to 0.85 [23,24].

Furthermore, we employed the Hospital Anxiety and Depression Scale (HADS) [11], used by health professionals to assess stress and depression levels. The HADS, also translated into Greek, is a scale of 14 elements; half of them pertain to stress and the other half to depression [25]. It has been reported to exhibit Cronbach’s alpha reliability ranging from 0.71 to 0.88 [26,27].

The participants were also asked questions regarding their department’s staffing adequacy and whether their frequency of workplace errors could be attributed to a potential shortage of staff.

### 2.4. Statistical Analysis

The statistical analysis was conducted with the 21st version of the Statistical Package for Social Sciences software (SPSS Statistics, IBM Corp., Armonk, NY, USA). The investigated variable was the potential incidence of workplace errors. We estimated the average and the standard deviation, regarding quantitative variables; conversely, both the count (N) and relative frequencies (%) of the values of the variables are presented, regarding qualitative variables. The chi-square test was used to assess the independence of each qualitative variable (sex, age, marital status, income, staffing of the department, professional burnout, and wellness).

Adjusted odds ratios and corresponding 95% confidence intervals (95% CIs) were calculated via multivariate logistic regression. Alpha level was set at 0.05 and an alpha level of 0.20 was used as the cut-off for variable removal in the automated model selection for multivariate logistic regression. Backward elimination proved more parsimonious and was used to assess the association between specific independent variables of the patients. The Type I error probability associated with all tests in this study was set to 0.05. Statistical analyses were performed using the statistical package for social sciences statistical software (version 24.0, IBM SPSS Statistics for Windows, Armonk, NY, USA: IBM Corp.).

## 3. Results

Participants’ sociodemographic characteristics (age, level of education, and family income) were recorded and are shown in Table 1.

The results pertaining to the relation between the demographic data and workplace errors showed that the only factor associated with errors was family income (Table 2). According to the analysis, 16.67% of the participants experienced depression, 50% experienced stress, 25% probably experienced depression, and 16.67% probably experienced stress (Table 3). Most participants had a high score in emotional burnout and depersonalization, and a low score in personal achievement. Only few participants (*n* = 16) did not experience professional burnout.

Almost all participants stated that their departments were understaffed, either because of vacant positions or because the workload of each employee was above average; only 10 employees stated that their department was adequately staffed.

The backward stepwise regression (Table 4) showed that the factors associated with workplace errors included inadequate staffing of the department (*p* < 0.05), wellness (*p* < 0.05), professional burnout (*p* < 0.05), and family income (*p* < 0.05).

Regarding the staffing of the department, the odds ratio (OR) (=1.818) showed that the relative probability of an error in understaffed departments was 81.8% higher in comparison with the departments that were adequately staffed. The 95% CI was 1.119–2.101 and was expected regarding the total population percentage. It is therefore considered accurate and reliable. Regarding wellness, the OR for individuals who scored low on wellness was 1.925; therefore, the probability of a workplace error was 92.5% higher than for individuals with a high level of wellness. The 95% CI was 1.122–3.303. The OR for professional burnout was 3.138. Consequently, the relevant probability of an error made by employees with burnout was 213.8% higher compared to employees who did not experience professional burnout. The 95% CI was 1.858–5.299. Regarding family income, it was found that the relevant probability of an error made by those who had an income of EUR 2000–EUR 3000 was 1332.2% higher than for those with an income of >EUR 3000.

## 4. Discussion

This study aimed to assess the relationship between professional burnout, wellness, and staffing and employee efficiency and workplace errors among the administrative staff of the Greek National Organization for the Provision of Health Services. Studies focusing on the psychological impact of the SARS and H1N1 pandemics on healthcare professionals have shown increased distress and depression symptoms [28,29]. Similar findings are reported by Preti et al. (2020) in the case of the COVID-19 pandemic [30]. This study recorded important levels of depression and stress among healthcare professionals in Greece, suggesting that health crises negatively influence the psychological state of healthcare workers. Furthermore, most participants experienced increased emotional burnout and depersonalization. Previous research has shown that professional burnout is not directly connected with workplace errors, even if depression is included in the analysis [17]; however, depression is recognized as an independent predictive factor of workplace errors. Conversely, studies using multifactorial analysis have reported that both burnout and wellness were independent predictive factors of an error in the workplace [31,32,33]. Holden et al. (2011) have also reported significant correlations between professional burnout and workplace errors [33]. Our results confirm these findings, showing a strong correlation (*p* < 0.00) between professional burnout and workplace errors.

Studies on burnout have concluded that more errors are essentially connected to physician fatigue [34,35], while other studies have shown a correlation between exhaustion and errors [36,37]. Specifically, Halbesleben et al. concluded that increased exhaustion is essentially connected to a lower frequency of the reported safety of the patient [36]. Klein et al. concluded that exhaustion is only associated with therapeutic (OR = 2.54) and diagnostic errors (OR = 1.94) in male, but not in female, surgeons in Germany [37]. This suggests that sex is a potential contributing factor to the prevalence of workplace errors. In this study, sex was not associated with workplace errors.

Brophy et al. report that understaffing due to prior financial problems and increased workload during health emergencies, and specifically the COVID-19 pandemic, affect the overall mental health of healthcare professionals, leading to exhaustion and burnout [38]. Our results showed a correlation between workplace errors and professional burnout, employee wellness, and inadequate staffing. Increased workloads and shortages in staffing were associated with the participants’ quality of work. The quality of work is particularly important for the medical, nursing, and administrative staff, including the employees in the auditing services and the staff councils; workplace errors in a hospital could result in human casualties, especially during the current pandemic. Therefore, particular attention should be paid to the human factor.

This study has some limitations. First, it included a relatively small sample of employees, which, however, comprises 50% of the total number of employees of the Departmental Boards of the Greek National Organization for the Provision of Health Services. Of course, it is very small given the Greek NHS’s overall employee number. Future studies might be conducted on a larger sample to ensure the generalizability of the results. Moreover, it employed a cross-sectional design. A longitudinal analysis is required to provide further insight and confirm our findings. To our knowledge, there is limited research regarding the recent COVID-19 pandemic’s influence on the prevalence of workplace errors among the administrative staff of the Greek National Organization for the Provision of Health Services, and our findings could serve as a basis for further investigation. Another pertinent limitation is that although medical, nursing, and administrative staff was included, a connection towards their responsibilities was not feasible because the questionnaires did not record this information as it would jeopardize anonymity.

As burnout sets in, healthcare workers may experience reduced concentration, diminished decision-making abilities, and decreased attention to detail. This can translate into an increased likelihood of workplace errors in tasks such as surgery, medication administration, and patient assessment. In the context of diseases of the head and neck, errors can have profound consequences, potentially impacting patient outcomes, exacerbating conditions, or causing unnecessary complications.

In conclusion, this study showed a strong association between burnout and workplace errors among healthcare professionals in Greece, with understaffed hospitals and increased pressure. Our results could contribute to the development of targeted interventions to support the mental health of healthcare staff and prevent incidents of post-traumatic stress [39,40]. In a context of constant understaffing and increased workload, avoiding workplace errors in the healthcare sector is imperative. Further measures should be taken to reinforce the staff in every department and to investigate the inadequate staffing of the departments, as there is a great possibility that it is not a factor that leads to expenditure savings; in contrast, it could lead to an increase in the cost of the provided services.

Implications for Occupational Health Practice: Our findings indicate a strong correlation between burnout and workplace errors among healthcare professionals; understaffed hospitals and increased pressure may contribute to increased workplace errors and, thus, lead to a reduced quality of healthcare services and increased costs. To endure optimal patient care in this specialized field, it might be appropriate to address healthcare worker burnout through proper support systems, workload management, and attention to their mental and emotional well-being.

Applying Research to Occupational Health Practice: We investigated the effects of wellness and burnout on workplace errors among the administrative staff in the Greek healthcare system. Questionnaires were administered to 120 participants; occupational burnout was assessed using the Maslach Burnout Inventory - Human Services Survey and the Hospital Anxiety and Depression Scale. Workplace errors were associated with inadequate staffing, a low sense of well-being, exhaustion, and low family income; a strong correlation was observed between professional burnout and workplace errors. Healthcare staff should be supported through targeted interventions focusing on mental health and staff shortages.

## Figures and Tables

**Table 1:** Breakdown of the Departmental Boards of the Greek National Organization for the Provision of Health Services employees survey sample by sex, age, level of education, and family income.

Sex	*N*	%
Men	46	38.33%
Women	74	61.67%
Age (years)		
<40	44	36.67%
>40	78	63.33%
Level of Education		
Technological Institute	20	16.66%
University	90	75.01%
Master/PhD	10	8.33%
Family Income (monthly)		
EUR 2000–3000	50	41.67%
EUR 3000–4000	60	50%
>EUR 4000	10	8.33%

**Table 2:** Relation of workplace errors to demographic characteristics in the Departmental Boards of the Greek National Organization for the Provision of Health Services employees survey sample.

	Chi Squared Statistic *	*p*-Value
Sex	0.574	0.464
Age	6.521	0.014
Education	0.000	1.000
Family income	20.353	<0.01

* Chi squared statistic in two-by-two tables between each variable and workplaces errors.

**Table 3:** Departmental Boards of the Greek National Organization for the Provision of Health Services employees survey sample participants’ mental state.

	Depression		Stress	
Cutoff Value	*n*	%	*n*	%
Actual (11–21)	16	16.67	50	50
Possible (8–10)	25	25	87	16.67
None (0–7)	79	58.33	33	33.33

**Table 4:** Departmental Boards of the Greek National Organization for the Provision of Health Services employees survey sample results of the backward stepwise model related to errors.

	Odds Ratios	Confidence Interval	*p*-Value
Adequate staffing	1.818	1.119–2.954	0.016
Wellness	1.925	1.122–3.303	0.017
Professional burnout	3.318	1.858–5.299	0.055
Family income			
EUR 3000–4000	14.322	1.911–107.346	0.010
>EUR 4000	8.531	0.953–76.070	0.005

Binary logistic regression with backward stepwise model elimination; odds ratios mutually adjusted for all variables included in the model (adequate staffing, wellness, professional burnout, family income).

## Data Availability

The data underlying this article shall be available on reasonable request to the corresponding author.
